# 

**Published:** 1866-12

**Authors:** 


### CLOSE OF THE VOLUME.

This number closes the twentieth volume of the Dental Register. The publisher thankful for the liberal support given to, and the appreciation manifested for the REGISTER, hopes for the future to make it more worthy of both. We presume none have a keener sense of its imperfections than we, and yet these have been of such a nature that we could not wholly control them; we hope in the future to render them less and less till they wholly disappear.

We send out bills with this number to all who are in arrears, and hope most earnestly to have a full response to every one. As we expend the entire income of the REGISTER on its publication, we see no good reason why we should advance the subscription of some of the readers of the REGISTER for from one to four years, in addition to devoting a large portion of time to editorial and publication work. We hope that the REGISTER will receive the hearty support of the profession, both with the few and many.

### THE TWENTY-FIRST VOLUME.

The January Number will introduce the twenty-first volume of this Journal. We are most happy to state that Dr. Watt has again consented to become one of the editors. We feel confident that this will afford the greatest pleasure to the readers of the REGISTER and meet with their highest approbation. Dr. W.'s abilities in the editorial chair are so well known to the profession that it is unnecessary for us to say aught upon the subject here. In this fact our readers have a guarantee of improvement in the journal. The forthcoming volume will not be much changed in appearance from the present, but improvements will be made just as rapidly as the *runner!* will warrant. If any desire to stop their subscription with the present volume, we hope they will notify us of the fact immediately, and if any wish to continue we are very anxious that they should notify us of their intention, by sending through the mail or otherwise, the insignificant sum of Three Dollars.

### PENNSYLVALIA COLLEGE OF DENTAL SURGERY.

THE ELEVENTH ANNUAL SESSION 1866–67.

### FACULTY.

J. D. WHITE, D.D.S., Emeritus Professor.
T. L. BUCKINGHAM, D.D.S., Prof. of Chemistry and Metallurgy.
E. WILDMAN, D.D.S., Prof. of Mechanical Dentistry.
G. T. BARKER, D.D.S., Prof. of Principles of Dental Surgery and Therapeutics.
W. S. FORBES, D.D S., Prof. of Anatomy and Physiology.
JAMES TRUMAN, D.D.S., Prof. of Dental Physiology and Operativ Dentistry.
EDWIN T. DARBY, D.D.S., Demonstrator of Operative Dentistry.
J. M. BARSTOW, D.D.S., Demonstrator of Mechanical Dentistry.

The regular Course will commence on the first Monday of November and continue until the first of March ensuing. During October the Laboratory will be open, and a Clinical Lecture delivered every Saturday, by one of the Professors, at 3 o'clock, P. M. The most ample facilities are furnished for a thorough course of practical instruction.

| | |
|---|---|
| Tickets for the Course, Demonstrators' Tickets included, | $100 |
| Matriculation Fee, - - - - - - - - | 5 |
| Diploma Fee, - - - - - - - - - - | 30 |

For further information address

T. L. BUCKINGHAM, Dean,
243 North Ninth Street, Philadelphia.

### NITROUS OXIDE.

### FOR DENTISTS.

By our new Self-Watching process, we guarantee an absolutely pure gas, without trouble, and at *half the usual cost.* Our Washers, Gasometer, and Inhaler, made on strictly scientific principles, are unique and beautiful. By this process the gas is given pure —no breath is inhaled. Of some 60 Dentists using our new process, not one has ever expressed any dissatisfaction. Agents in Cincinnati and St. Louis wanted. Send for circular.

**A. W. SPRAGUE, Chemist,**

*89 Washington St., Boston.*

### BALTIMORE COLLEGE OF DENTAL SURGERY.

TWENTY-SIXTH ANNUAL SESSION, 1866–67.

---

### FACULTY.

THOMAS E. BOND, A.M., M.D., Prof. of Pathology and Therapeutics.
PHILIP H. AUSTEN, A.M., M.D., D.D.S., Prof. of Dental Science and Mechanism.
FERDINAND J. S. GORGAS, A.M., M.D., D.D.S., Prof, of Dental Surgery
A. SNOWDEN PIGGOT, A.M., M.D., Prof. of Chemistry and Metallurgy.
RUSSELL MURDOCH, A.M., M.D., Prof. of Anatomy.
HENRY REGINALD NOEL, A. M., M. D., Professor of Physiology.
HENRY HOBART KEECH, D.D.S., Demonstrator of Operative Dentistry.
THOMAS SOLLERS WATERS, D.D.S., Demonstrator of Mechanical Dentistry.
CLAUDE BAXLEY, M. D., Demonstrator of Anatomy.

The *Twenty-Seventh* Annual Session will commence on the first of November, 1866, and close on the first of March, 1867.

| | |
|---|---|
| Lecture and Demonstration Fees, - - - | $115 |
| Matriculation Fee (paid only once) - - - - | 5 |
| Diploma Fee. - - - - - - - | 30 |

For information, address

F. J. S. GORGAS, M.D., Dean of the Faculty.

---

### CHARLES ABBEY & SONS

MANUFACTURERS OF

### DENTISTS' FINE GOLD AND TIN FOIL,

***Nos. 228 and 230 Pear St.,***

PHILADELPHIA.

The attention of Dentists is invited to our FINE GOLD FOIL, which is prepared under our constant personal supervision. Our Nos. are 4, 5, 6, and 8.

We are also manufacturing an ADHESIVE FINE GOLD FOIL, Nos. 4 5 and 6.

ALL our Gold Foil is manufactured from ABSOLUTELY PURE GOLD, prepared expressly for the purpose with great care, by ourselves.

### Dentist's Refined Tin Foil constantly on hand

CHARLES ABBEY,

WM. R. ABBEY,

CHAS. O. ABBEY.

### NEW YORK
### COLLEGE OF DENTISTRY.

### FACULTY.

ELEAZAR PARMLY, M. D., D. D. S.,
Emeritus Professor of the Institutes of Dentistry.

J. SMITH DODGE, Jr., M. D. D. D. S.,
Professor of Dental Pathology and Therapeutics.

WM. H. DWINELLE, M. D., D. D. S.,
Professor of Dental Science and Operative Dentistry.

FANEUIL D. WEISSE, M. D.
Professor of Descriptive and Comparative Anatomy.

NORMAN W. KINGSLEY,
Professor of Dental Art and Mechanism.

RUFUS KING BROWNE, M. D.,
Professor of Experimental Physiology and Microscopy.

CHARLES A SEELY, A. M.,
Professor of Chemistry and Metallurgy.

### DEMONSTRATORS.

D. H. GOODWILLIE, M. D., D.D.S.

Operative Dentistry.

R. M. STREETER,

Mechanical Dentistry.

ALEX. W. STEIN, M. D., Anatomy.

### BOARD OF CLINICAL LECTURERS.

DR. JOHN ALLEN, New York, *President.*

Dr. EHRICK PARMLY, N. Y.

" J. D. WHITE, Philadelphia.

" WM. H. ALLEN, New York.

" I. J. WETHERBEE, Boston.

" W. B. ROBERTS, New York.

" C. A. MARVIN, Brooklyn.

" A. C. HAWES, New York.

" A. McILROY, New York.

Dr. W. W. ALLPORT, Chicago.

" GEO. E. HAWES, New York.

" A. HILL, Norwalk, Conn.

" J. N. H. WALBRIDGE, N. Y.

" CHAS. B. FOSTER, Utica.

" L. G. BARTLETT, New York.

" J. T. METCALF, New Haven.

" R. W. VARNEY, New York.

Dr. FRANK ABBOTT, N. Y., *Secretary.*

The plan of organization of this College provides for a course of instruction, didactic and demonstrative, by the above-named Faculty and their Demonstrators, in Practical Dentistry and collateral sciences; and also a Dental Infirmary, where clinical instruction will be given by the Board of Clinical Lecturers, which comprises a large number of the most skillful gentlemen in the profession throughout the country. This Board will give daily Clinics during the entire session. The advantages to the student of such a combination in acquiring a thorough and practical knowledge of the science and art, it is believed, cannot be excelled. The session will open on Monday, November 5th, 1866, and continue until the 1st of March following.

Tickets for the course, $150, Matriculation, Demonstrators, and diploma fees included. For further information, see College Announcement, or address the Dean of the Faculty. NORMAN W. KINGSLEY, Dean,
*No. 25, West 27th St., New York.*

LEEAZAR PARMLY,

*Pres't Board of Trustees.*

### PHILADELPHIA DENTAL COLLEGE

108 North Tenth St., above Arch.

FOURTH ANNUAL SESSION, 1866-67.

### TRUSTEES.

PRESIDENT.
REV. RICHARD NEWTON, D.D.

SECRETARY.
R. SHELTON MACKENZIE, D.C.L.

PETER F. ROTHERMEL,

WILLIAM DULTY,

GEORGE J. ZIEGLER, M.D.,

GEORGE WILLIAMS,

ROBERT L. McCLELLAN, D.D.S.

J. L. SUESSEROTT, M.D., D.D.S..

COLSON HIESKELL,

S. FISHER CORLIES,

JAMES L. CLAGHORN,

EX-GOV. JAS. POLLOCK,

CHARLES S. BECK, M.D.

HENRY CRUMLEY.

### FACULTY

C. A. KINGSBURY, D.D.S.,
Emeritus Professor of Dental Physiology and Operative Dentistry.

HENRY MORTON, A.M.,
Emeritus Professor of Chemistry.

GEO. W. ELLIS, D.D.S.,
Professor of Dental Physiology and Operative Dentistry

THOS. WARDLE, D.D.S.,
Professor of Mechanical Dentistry and Metallurgy.

J. H. McQUILLEN, D.D.S.,
Professor of Anatomy, Physiology and Hygiene.

J. FOSTER FLAGG, D.D.S.,
Professor of Institutes of Dentistry.

ALBERT R. LEEDS, A. M..
Professor of Chemistry.

T. C. STELLWAGEN, D.D.S.,
Demonstrator of Operative Dentistry

WM. P. HENRY, D.D.S.,
Demonstrator of Mechanical Dentistry.

The Dispensary and Laboratory of the College will be open, and preliminary lectures will be delivered by one of the Professors every day during the latter half of October; commencing on the 17th instant, the lecture on Wednesday of each week, at 3 o'clock P.M., to be devoted to *Clinical* teaching. The regular Course of instruction will commence on the first Monday of November, and continue until the close of the ensuing February.

The lectures will be amply illustrated by the extensive and *valuable* collections of Anatomical, Pathological, and Mineralogical specimens, and the Philosophical and Chemical apparatus of the incumbents of the various Chairs, and every opportunity will be afforded in the Clinic and Laboratory for obtaining a *practical* knowledge of Operative and Mechanical Dentistry.

### FEES.

| | |
|---|---|
| Matriculation (paid but once) - - - - - - | $5 00 |
| Tickets for the Course, including the Demonstrators' - | 100 00 |
| Diploma - - - - - - - - - - | 30 00 |

For further particulars, address J. H. McQUILLEN, DEAN,
1112 *Arch Street, Philadelphia.*

y '66.

### OHIO COLLEGE
OF
### DENTAL SURGERY.

The Twenty-first Annual Session of the Ohio College of Dental Surgery will commencing on Monday the 29th of October, and continue to the first Wednesday of March succeeding.

### FACULTY.

JAMES TAYLOR, D. D. S.,
Emeritus Professor of Institutes of Dental Science.

C. W. SPALDING, D. D. S.,
Professor of Special Histology, Physiology and Pathology.

C. KEARNS, M. D.,
Professor of Anatomy and Physiology.

H. R. SMITH, D. D. S.,
Professor of Mechanical Dentistry and Metallurgy.

GEORGE WATT, D. D. S.,
Professor of Dental Chemistry and Therapeutics,

J. TAFT, D. D. S.,
Professor of Operative Dentistry and Dental Hygiene.

C. R. BUTLER, D. D. S.
Professor of Clinical Dentistry.

WILL TAFT, D. D. S.
Demonstrator of Anatomy.

### TEXT BOOKS.

Gray's and Wilson's Anatomy; Dalton's or Carpenter's Physiology; Williams' Pathology; Fown's, Turner's, Graham's, or Brandt and Taylor's Chemistry; Taft's Operative Dentistry; Richardson's Mechanical Dentistry; Harris' Principles and Practice of Dental Science, Tomes' Dental Surgery.

### TERMS OF ADMISSION.

Tickets must be procured at the beginning of the session.

| | |
|---|---|
| Tickets for the course, | $100 00 |
| Demonstrator's Ticket for Anatomy, | 5 00 |
| Matriculation Fee, but once, | 5 00 |
| Diploma Fee, | 30 00 |

### TERMS OF GRADUATION.

Two courses, (including one of dissections,) with a satisfactory dental pupilage.

Four years reputable practice will be received as equal to one course.

An acceptable thesis upon some subject on Dental science.

Must possess a good moral character, and be twenty-one years old.

For further information, address J. TAFT, *Dean.*
172 Race St.

### CONTENTS.

### OF THE WEST.

*Issued Monthly, at $3 a Year in Advance.*

DEVOTED TO THE INTERESTS OF THE DENTAL PROFESSION.

**EDITED BY J. TAFT**

The volume commences in January and closes in December.

The Register being issued monthly is always fresh, therefore indispensable to the practitioner who desires to keep posted up with the new inventions and improvements which are daily developed. Neither expense nor effort will be spared to give value and variety to its contents. Articles will be illustrated with well executed engravings whenever they will add to the interest or assist in explanation.

Its extensive circulation and frequency of issue makes it one of the BEST DENTAL ADVERTISING MEDIUMS in the United States.

RATES OF ADVERTISING.

One page, 1 year, $50. Half page, 1 year, $30. Quarter page, or card, 1 year $20.

All advertising bills payable in advance, or at furthest after the issue of the first number containing the advertisement. All transient advertisements to be paid for in advance.

☞ CONTRIBUTIONS SOLICITED.

Address, J. TAFT, or "DENTAL REGISTER," Cincinnati, Ohio.

Business Communications to

**J. TAFT, Publisher,**
No. 238 Fourth St., between Plum and Central Avenue.

